# Cortisol Immunosensors: A Literature Review

**DOI:** 10.3390/bios13020285

**Published:** 2023-02-16

**Authors:** Chrysoula-Evangelia Karachaliou, Georgios Koukouvinos, Dimitrios Goustouridis, Ioannis Raptis, Sotirios Kakabakos, Panagiota Petrou, Evangelia Livaniou

**Affiliations:** 1 Immunopeptide Chemistry Lab., Institute of Nuclear & Radiological Sciences & Technology, Energy & Safety, National Centre for Scientific Research ‘‘Demokritos”, P.O. Box 60037, 153 10 Agia Paraskevi, Greece; 2 Immunoassay/Immunosensors Lab., Institute of Nuclear & Radiological Sciences & Technology, Energy & Safety, National Centre for Scientific Research ‘‘Demokritos”, P.O. Box 60037, 153 10 Agia Paraskevi, Greece; 3 ThetaMetrisis S.A., Christou Lada 40, 121 32 Athens, Greece; 4 Department of Electrical & Electronics Engineering, University of West Attica, 122 44 Athens, Greece; 5 Institute of Nanoscience and Nanotechnology, National Centre for Scientific Research ‘‘Demokritos”, P.O. Box 60037, 153 10 Agia Paraskevi, Greece

**Keywords:** biomarkers, biological samples, blood plasma/serum, cortisol, electrochemical immunosensors, immunoassays, optical immunosensors, saliva, sweat

## Abstract

Cortisol is a steroid hormone that is involved in a broad range of physiological processes in human/animal organisms. Cortisol levels in biological samples are a valuable biomarker, e.g., of stress and stress-related diseases; thus, cortisol determination in biological fluids, such as serum, saliva and urine, is of great clinical value. Although cortisol analysis can be performed with chromatography-based analytical techniques, such as liquid chromatography–tandem mass spectrometry (LC-MS/MS), conventional immunoassays (radioimmunoassays (RIAs), enzyme-linked immunosorbent assays (ELISAs), etc.) are considered the “gold standard” analytical methodology for cortisol, due to their high sensitivity along with a series of practical advantages, such as low-cost instrumentation, an assay protocol that is fast and easy to perform, and high sample throughput. Especially in recent decades, research efforts have focused on the replacement of conventional immunoassays by cortisol immunosensors, which may offer further improvements in the field, such as real-time analysis at the point of care (e.g., continuous cortisol monitoring in sweat through wearable electrochemical sensors). In this review, most of the reported cortisol immunosensors, mainly electrochemical and also optical ones, are presented, focusing on their immunosensing/detection principles. Future prospects are also briefly discussed.

## 1. Introduction

The steroid hormone cortisol is the final product of the well-known hypothalamic–pituitary–adrenal (HPA) axis, one of the four major neuroendocrine systems through which the hypothalamus and pituitary gland govern neuroendocrine function. Cortisol is synthesized in the zona fasciculata/reticularis cells of the adrenal cortex, under the control of adrenocorticotropic hormone (ACTH), and then secreted to the circulation. Approximately 80–90% of cortisol in blood plasma is bound to corticosteroid-binding protein (CBP), the rest is circulated bound to albumin, and only a small fraction, <10%, of total hormone is free circulating cortisol. Cortisol is lipophilic enough to cross the plasma membrane of target cells without the need of a membrane transporter. Within the target cell, cortisol binds to intracellular glucocorticoid receptors and the complexes formed translocate to the nucleus, where they may either enhance or decrease the expression of multiple genes. In this way, cortisol may affect many important biochemical functions, including release of ACTH, synthesis of gluconeogenesis-related enzymes, breakdown of muscle proteins, and lipolysis. Cortisol may be transformed into its inactive metabolite, cortisone, either reversibly through the action of 11β-hydroxysteroid dehydrogenase (11β-HSD1), an enzyme expressed mainly in the liver as well as in the subcutaneous and visceral adipose tissue, or irreversibly through the action of 11β-hydroxysteroid dehydrogenase isozyme (11β-HSD2), which is expressed mainly in the adrenal cortex, as well as in the renal distal tubules and collecting ducts. Cortisol is removed from the body through the liver and kidneys [1,2].

Cortisol concentrations in the body are a valuable indicator of a series of important activities and functions of the organism crucial for homeostasis, and may also serve as a biomarker in a variety of disorders [2](2. Boolani_2019). More specifically, cortisol levels have been investigated in/correlated with a series of stress-associated disorders [3,4,5], while they are considered a hallmark in the diagnosis of Cushing’s syndrome (neoplastic hypercortisolism) [6]. Thus, the 2008 Endocrine Society clinical practice guidelines have recommended measurement of cortisol levels under precisely defined conditions (i.e., late-night salivary cortisol, 24 h urinary free cortisol (UFC) and serum cortisol after dexamethasone-suppression test, as screening tests for Cushing’s syndrome [1,7,8]. Moreover, monitoring of cortisol levels has been considered a useful approach to detect recurrences in patients who have undergone pituitary surgery [6] or to investigate so-called adrenal incidentalomas/adrenal insufficiency [9].

As with many other hormones, cortisol shows diurnal variation. More specifically, its levels start to increase between 03:00 h and 04:00 h, reach a peak between 07:00 h and 09:00 h, and then fall steadily, with the lowest level (nadir) observed at about midnight [7]. Thus, collection schedules of the biological fluids to be analyzed should be strictly planned.

Cortisol is most often determined in blood serum/plasma, saliva, or urine. As already mentioned, the vast majority of circulating cortisol is protein-bound, mainly to CBP and to a lesser extent albumin, while only a small fraction occurs in free form. [1,2]. According to the free hormone hypothesis, the level of unbound hormone determines its biological activity, and thus measurement of free serum/plasma cortisol is of the greatest clinical value [1]. However, measurement of free cortisol in serum/plasma samples requires an often time-consuming and laborious preanalytical step, usually including equilibrium dialysis, gel filtration or ultrafiltration, to separate free from bound cortisol, and is therefore difficult to perform in routine clinical practice [1]. Instead, total (bound and unbound) cortisol is usually determined. Serum cortisol levels of normal individuals have been reported to range between 45 and 227 ng/mL in the morning and 17 and 141 ng/mL in the evening [10].

Measurement of cortisol levels in saliva is considered a reliable alternative to measuring free cortisol in serum/plasma, since there is a high correlation between salivary cortisol levels and unbound cortisol levels in serum/plasma; thus, salivary cortisol is believed to reflect the changes in free serum/plasma cortisol [2,11,12,13]. Saliva is considered a body fluid with well-recognized diagnostic potential [14], while saliva sampling has the advantage of causing the least possible intrusion to the organism, thus avoiding stress, which may in turn affect cortisol levels [2]. To this end, as already mentioned, late-night salivary cortisol has been recommended as a first-line screening test for the diagnosis of Cushing’s syndrome [1]. To avoid critical changes in the oral milieu that may affect measurement, sampling instructions should be strictly followed. Thus, use of ointments and creams containing hydrocortisone (chemically identical with cortisol) should be avoided before sampling, since it may lead to false-positive results due to contamination of the saliva sample [6,15]. Moreover, various factors, such as age, gender, medical status/medications, menstrual cycle, pregnancy, caffeine, and alcohol consumption, should be taken into consideration before evaluating the clinical meaning of cortisol measurement in saliva [2,16,17]. Under normal conditions, saliva cortisol levels have been reported to range between 1 and 12 ng/mL in the morning and 0.1 and 3.0 ng/mL in the evening [10].

A small fraction of free circulating cortisol (1–2%) is excreted in urine, and urine cortisol levels are also believed to reflect the levels of free cortisol in serum/plasma. Urine cortisol exists in conjugated (e.g., glucuronide-conjugated and sulfate-conjugated) and unconjugated (free) forms. Measurement of 24 h UFC, which is not affected by diurnal variation, is considered to reliably reflect tissue exposure to free cortisol over a day, and has therefore been used as a well-established screening test for the diagnosis of Cushing’s syndrome, as already mentioned [1,7,8]. Under normal conditions, urine cortisol levels have been reported to range between 21.5 and 149.7 μg/24 h [10] or between 10 and 100 μg/24 h [18].

Besides determination of cortisol levels in blood serum/plasma, saliva, or urine samples, measurement of cortisol extracted from human hair (usually, 1–3 cm of hair from the posterior vertex region of the scalp) has also been reported [19,20]. Since hair has been reported to grow at an average rate of 1 cm per month [21], hair samples allow determination of cumulative cortisol over a period of several months. In this regard, hair cortisol levels have been suggested as a retrospective, long-term biomarker of HPA axis functioning [20], stress-related, emotional and behavioral symptoms [22,23], or systemic exposure to cortisol in patients with Cushing’s syndrome [1]. Hair cortisol levels have been reported to range between 18 and 153 pg/mg [10] and 1.7 and 153.2 pg/mg [18]. In addition, cortisol levels in fingernail samples may also serve as a putative retrospective biomarker [24,25]. Finally, measurement of cortisol metabolites in feces of animals has been used as a noninvasive means to evaluate release of glucocorticoids and thus adrenocortical activity in various animal species [26].

The two main methods for measuring cortisol in biological samples are immunoassays and LC-MS/MS [2,7,27,28,29].

Cortisol immunoassays are based on antibodies recognizing cortisol and are the most frequently used methods for determining cortisol in routine clinical practice. Cortisol immunoassays include radioimmunoassays and ELISAs [12], as well as chemiluminescent immunoassays, electrochemiluminescence immunoassays, and fluorescence immunoassays [2,7]. In most cases, immunoassays are the current method of choice to determine total (bound and unbound) cortisol in serum/plasma as a fast and reproducible approach to estimate cortisol status; however, immunochemical determination of total cortisol in serum/plasma samples may be affected by changes in CBP and/or albumin levels [1,30]. Immunoassays are also the most frequently used method for determining urine free (unconjugated) cortisol (UFC) in clinical laboratories. Moreover, immunoassays have been widely applied to the determination of salivary cortisol, since they can provide the low detection limits required to quantitate the hormone present in the salivary samples, even at the nadir of the diurnal rhythm [1].

Despite their wide use, the analytical specificity of cortisol immunoassays may be limited by antibody cross-reactivity with other steroids present in the sample, while preanalytical interferences may also occur [15]. On the other hand, LC-MS/MS methods are characterized by high specificity and are progressively more and more often used in clinical laboratories [1], especially when interferences are suspected to occur [9]. Nevertheless, immunoassays are still preferred for cortisol determination and are considered the method of choice for assessing serum/plasma cortisol levels as a fast screening, especially in emergency cases.

Immunosensors are advanced analytical platforms, which—similarly to conventional immunoassays—are based on antibodies recognizing the analyte of interest [31]. Immunosensors exhibit a series of advantages in comparison with immunoassays, such as simple and fast assay protocols, which can be performed by nonexperts, as well as small detection modules, which allow their broader application, i.e., even outside the lab. Thus, the development of antibody-based sensors, especially at the point of care, and/or wearable sensors that would allow real-time cortisol monitoring in different body fluids, including sweat [32], has been receiving increasing attention as a powerful alternative to conventional immunoanalytical methods. Due to the continuously growing interest in the field, we considered it worthwhile presenting an updated review on reported cortisol immunosensors for the analysis of different biological samples.

## 2. Immunosensors for Detecting Cortisol in Biological Samples

Cortisol immunosensors were first reported in the mid-1990s. One of the first cortisol immunosensors was a noninvasive, reusable amperometric immunosensor, which was based on an anti-cortisol antibody for biorecognition and horseradish peroxidase (HRP) for signal generation. The antibody and the enzyme were co-immobilized on a chemically activated membrane, which had been mounted around the tip of an oxygen electrode. The assay principle was that the current created in the presence of the enzyme substrate was reduced upon binding of the antigen to the co-immobilized antibody [33]. At about the same time, the development of an invasive cortisol immunosensor was reported [34]. That sensor employed an HRP-labeled cortisol conjugate and an anti-cortisol antibody immobilized on the surface of a platinum electrode (working electrode). The platinum electrode was inserted into commercially available microdialysis probes and the probes were modified so as to bear the reference (Ag/Cl) and counter (Ag) electrodes on their top and then implanted in the jugular vein and used for real-time determination of cortisol in conscious animals, by monitoring peroxidase activity [34]. Since then, many cortisol immunosensors—mostly noninvasive—have been reported in the literature. Cortisol immunosensors reported thus far are mainly electrochemical and optical, while one of the first immunosensors described for cortisol was piezoelectric [35].

Two previous review articles have provided accumulated information on immunosensors, both electrochemical and optical ones, for determining cortisol—usually among other health biomarkers—focusing on the analysis of saliva samples [28,36]. An informative table summarizing reports on various cortisol sensors based on different detection techniques was included in a recent article [37]. However, the information currently available in the literature might be considered, at least in our opinion, somewhat “fragmented.”

### 2.1. Electrochemical Cortisol Immunosensors

The first cortisol immunosensors, reported in the mid-1990s [33,34] as already mentioned, were electrochemical. In the meantime, many more electrochemical immunosensors for cortisol have been described in the literature, while some recent review articles have provided accumulative information on cortisol electrochemical sensors [10,38].

During the last decade, various wearable electrochemical sensing platforms, which in general are considered ideal for analyzing sweat samples [39], have been developed and applied to the immunodetection of cortisol in sweat, as critically presented in previous review papers [18,40]. Several electrochemical cortisol immunosensors for sweat analysis have been described in the literature during the last couple of years (2020–2022) [41,42,43,44,45,46]. Tear analysis by means of electrochemical cortisol immunosensing has also been reported [47].

In this work, we present most of the articles published in recent decades regarding the development of electrochemical immunosensors for the detection of cortisol in a variety of samples, ranging from plain buffer solutions [33,48,49] to complex biological specimens. As shown in Table 1, electrochemical cortisol immunosensors have been mainly applied to the analysis of saliva samples [37,50,51,52,53,54,55,56,57,58,59,60,61,62,63], and to a lesser extent blood plasma or serum [64,65,66,67,68,69,70], interstitial fluid [63,71], buffer solutions of rat adrenal gland acute slices [58], and whole-body zebra fish [51]. Early electrochemical immunosensors applied to detect cortisol in vivo in dialysates of the extracellular fluid of animal brain (amygdala region) or in dialysates of circulating blood in conscious animals are also listed in Table 1 [34,72]. Moreover, as mentioned, cortisol electrochemical immunosensors have been employed as a means to analyze human/artificial sweat samples [41,42,43,44,56,73,74,75,76]. The underlying immunoassay principle and the electrochemical detection/signal principle of each sensor have been included in Table 1, while cortisol concentrations corresponding to the working range and/or limit of detection (LOD) have been listed as well.

**Figure 1:**
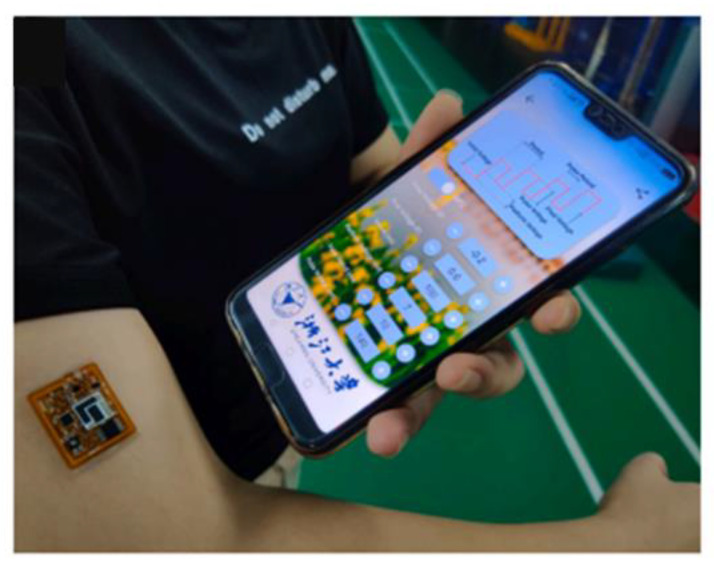
Real-time cortisol detection in human sweat with a wearable electrochemical immunosensor. Data were collected and displayed through a near-field communication–enabled smartphone (adopted with permission from [42]).

Among the most recently reported electrochemical cortisol immunosensors are the following (Table 1): (a) an electrochemical immunosensor for sweat cortisol based on an L-cys/-gold nanoparticles/-MXene(titanium carbide)–modified thread electrode [41]; (b) a photoelectrochemical immunosensor for salivary cortisol based on the competition between free cortisol and a bovine serum albumin (BSA)–cortisol conjugate immobilized onto magnetic beads for binding to an anti-cortisol antibody labeled with silver nanoclusters (AgNCs); after formation of the immunocomplexes, the magnetic beads were separated, the AgNCs were dissolved in nitric acid and the silver ions produced were transferred to the sensor electrode for ion exchange with cadmium sulfide (CdS) quantum dots, which led to a decrease in the photocurrent intensity [50]; (c) a battery-free, wireless and flexible electrochemical patch-type immunosensor for sweat cortisol determination employing near-field communication with a smartphone (Figure 1); the sensor employed an anti-cortisol antibody covalently immobilized on screen-printed electrodes, coated with gold nanoparticles (AuNPs) through a bifunctional polyethylene glycol (PEG) derivative [42]; (d) an electrochemical immunosensor for sweat cortisol based on a flexible electrode that was developed by transferring a multiwalled carbon nanotube (MWCNT) film on a polydimethylsiloxane substrate and subsequently depositing gold nanoparticles on the MWCNT surface [43]; (e) an electrochemical impedance spectroscopy immunosensor for detecting cortisol as a stress marker in saliva of trainee guide dogs during the training process [37]; (f) an immunosensor for salivary cortisol based on a gold nanoparticle–molybdenum disulfide–gold nanoparticles scaffold as transducer combined with a smartphone-operated point-of-care miniaturized differential pulse voltammetry system [52]; (g) an immunosensor employing a modified tin-doped indium oxide (ITO) electrode, on which an anti-cortisol antibody labeled with ferrocene tags had been immobilized; a point-of-care electrochemical platform was thus constructed and applied to determining cortisol in artificial saliva and whole-body zebrafish with or without extraction [51]; (h) an immunosensor using an integrated wireless sensing device that was based on laser-engraved graphene electrodes for detecting sweat cortisol; the cortisol diurnal cycle in human sweat was investigated with that immunosensor and a strong empirical correlation between serum and sweat cortisol was reported [44]; (i) a dual amperometric immunosensor microchip for the simultaneous detection of cortisol and insulin in untreated serum samples; insulin detection was based on a peroxidase-labeled sandwich assay, and cortisol detection on an alkaline phosphatase-labeled (ALP-labeled) competitive immunoassay [64].

Most of the electrochemical cortisol immunosensors are characterized by very good analytical features, such as high sensitivity, with LOD values in the range of pg/mL or even fg/mL, as shown in Table 1. Immunosensor specificity is mainly dependent on the anti-cortisol antibody employed, while other sensor reagents/components along with the biological matrix of the sample analyzed may also affect specificity, at least to some extent. For several electrochemical immunosensors [41,53,54,57], high reproducibility has been reported. However, larger validation studies are required before further sensor exploitation, as we mention in Section 3.

### 2.2. Optical Cortisol Immunosensors

The first optical cortisol immunosensors were developed in the late 2000s (Table 2). These sensors, based on the surface plasma resonance (SPR) detection principle [77,78,79] and applied to the detection of salivary cortisol, are mentioned in a recent review article concerning analysis of saliva as an ideal “health mirror” sample [80]. Since the late 2000s, several other optical cortisol immunosensors have been described in the literature.

In this work, we present most of the articles published in the last 15 years regarding the development of optical immunosensors for detecting cortisol in a variety of matrices, from plain buffer solutions [81,82,83] to complex biological specimens. As shown in Table 2, complex biological samples analyzed for cortisol with optical immunosensors include mainly saliva [78,79,84,85,86,87,88,89,90], as well as blood plasma/serum [91,92] and urine [78].

**Figure 2:**
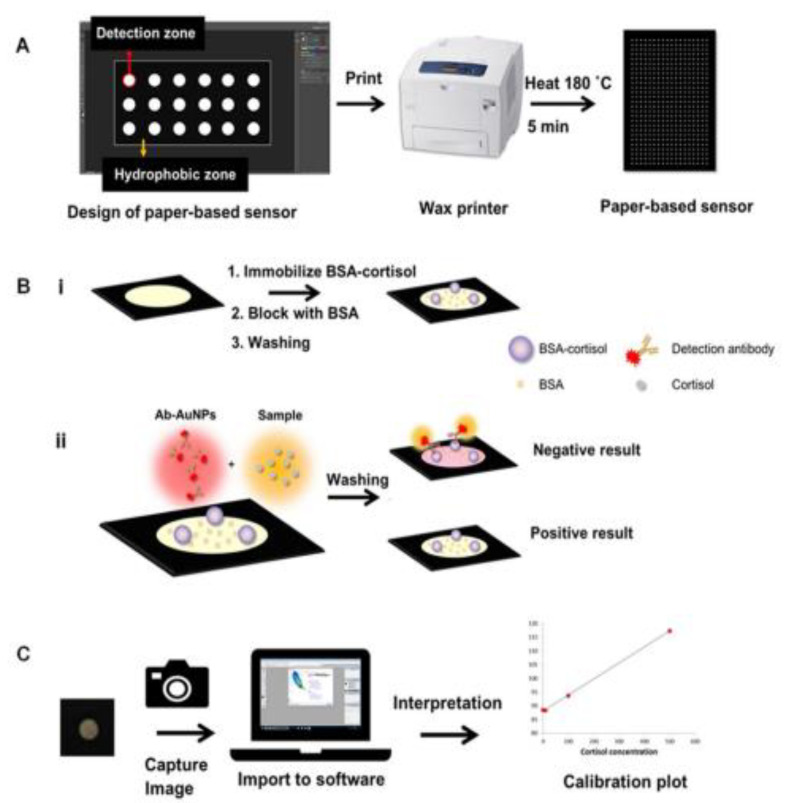
Schematic representation of: (**A**) production of paper-based sensors using a wax printer; (**B**, i) immobilization of the BSA–cortisol conjugate on the detection surface; (**B**, ii) competitive assay between cortisol in the sample and BSA–cortisol immobilized on the detection surface of the paper sensor for binding to an anti-cortisol antibody labeled with gold nanoparticles; (**C**) collection and interpretation of the results using image capture/processing programs. (Adopted with permission from [92]).

Among the most recently reported (2020–2022) optical cortisol immunosensors are the following (Table 2): (a) an optical immunosensor for real-time/continuous monitoring of cortisol in human blood plasma obtained after filtration or through microdialysis employing a complex immunoassay setup for cortisol biosensing through particle mobility monitoring with the aid of a microscope [91]; (b) an SPR immunosensor based on D-shaped optical fibers; the sensor has been applied to determining cortisol in buffer solutions [81]; (c) an SPR immunosensor based on an unclad plastic optical fiber, first coated with gold/palladium alloy and subsequently loaded with an anti-cortisol antibody; this sensor has also been applied to determining cortisol in buffer solutions [82]; (d) an optical immunosensor based on metal (gold)-enhanced time-resolved fluorescence for the continuous real-time monitoring of cortisol in buffer solutions [83]; (e) a paper-based optical immunosensor for serum cortisol, based on a competitive assay principle and employing an anti-cortisol antibody labeled with gold nanoparticles (Figure 2) as signal indicator [92]; (f) an optical immunosensor for salivary cortisol based on fluorescence quenching caused by cortisol binding to quantum dots loaded with an anti-cortisol antibody (or with an aptamer) [84].

Although optical cortisol immunosensors may be considered less sensitive than electrochemical ones, at least on average, most of them are characterized by adequate sensitivity, with LOD values mainly in the range of ng/mL, as shown in Table 2. Integration of advanced nanomaterials, especially during the last few decades, has led to signal enhancement and contributed to achieving increased analytical sensitivity. Similarly to electrochemical immunosensors, specificity of the optical cortisol immunosensors is mainly dependent on the specificity of the primary antibody employed, while it might also be affected by other reagents/components used for constructing the immunosensing platform and/or matrix of the biological samples. For some of the optical immunosensors [81], high reproducibility has been reported. Nevertheless, as in the case of electrochemical sensors, large validation studies are required before reaching a solid evaluation.

### 2.3. Cortisol Aptasensors and MIP-Based Biosensors

Aptamer-based biosensors are well-known alternatives to immunosensors and have been proposed for various applications in biomedical analysis [93,94]. Concerning cortisol, several biosensors based on a proper aptamer [95], instead of an anti-cortisol antibody, have been reported in the literature: similar to immunosensors, cortisol aptasensors are mainly electrochemical [96,97,98,99,100,101] and optical, including lateral flow–type sensing strips [84,102,103,104].

Biosensors based on molecularly imprinted polymers (MIP), instead of specific antianalyte antibodies, are another group of cortisol sensors [105,106]. Mainly electrochemical [107,108,109,110] and optical [111] MIP-based cortisol biosensors have been reported in the literature.

## 3. Discussion—Future Perspectives

Cortisol homeostasis is essential for human health, and abnormal cortisol levels have been correlated with and may serve as a valuable biomarker for several disease states. Thus, it is important to monitor cortisol concentrations in various biological samples by means of proper analytical methods. Cortisol immunoassays are currently considered the analytical method of choice for determining cortisol in biological specimens, such as blood serum/plasma, urine, saliva, or, more recently, hair. Transformation of the conventional immunoassays to technologically advanced antibody-based assays, which can easily be accomplished in a short time by unskilled persons and are capable of being “integrated” into portable devices for point-of-care measurements, has led to the development of several cortisol immunosensors during the last three decades. Cortisol immunosensors can be divided according to the signal transduction principle they rely upon. Most cortisol immunosensors are electrochemical and rely on signal measurement through cyclic voltammetry, impedance spectrometry, and amperometry. On the other hand, several optical immunosensors, including flow lateral–type strip sensors, have been developed, especially during the last decade. Besides the usually measured optical signals, e.g., SPR signals, some of the most recently reported cortisol immunosensors [91] rely on the measurement of other parameters, e.g., on particle mobility monitoring with the aid of a microscope [112].

Some immunosensors can simultaneously detect cortisol along with another biomarker, e.g., insulin [64], lactate [56] or IL-6 [75]. This “multianalyte” approach, although technologically difficult to achieve and therefore rarely reported, is highly desirable from a clinical point of view. Thus, a dual electrochemical immunosensor proposed for the simultaneous detection of cortisol and insulin at the point of care [64] may eventually offer improved management of diabetes.

A great number of cortisol immunosensors have been applied to the analysis of saliva samples and less to urine or blood plasma/serum, while too little information concerning immunosensors for hair cortisol is currently available, at least to our knowledge. This tendency might be attributed, at least partly, to special requirements for the collection and/or treatment of the relevant samples, e.g., the requirement of 24 h urine collection renders urine samples weak candidates for real-time detection of cortisol through a point-of-care immunosensor device [40]. Other factors supporting this trend may include matrix complexity, the need for careful pH adjustment before sample analysis, etc. On the other hand, a special group of electrochemical immunosensors (e.g., miniaturized and flexible/wearable sensors that are based on new materials, such as two-dimensional nanosheets of MoS_2_ [74]) have allowed real-time and even continuous monitoring of cortisol in sweat. As reported, free (protein-unbound) cortisol seems to be present in sweat glands, through a mechanism resembling transportation of free cortisol by the bloodstream to the salivary glands. From sweat glands, cortisol is thought to reach sweat by passive transportation through the cell lipid bilayer membrane [113]. Sweat cortisol concentrations have been reported to range from 8 to 142 ng/mL [10,18], the highest levels being found in the morning and correlating with salivary levels [113]. So far, sweat cortisol has not been detected with conventional methods—at least, not routinely—possibly due to difficulties in collecting and properly storing the corresponding samples for subsequent laboratory analysis.

**Figure 3:**
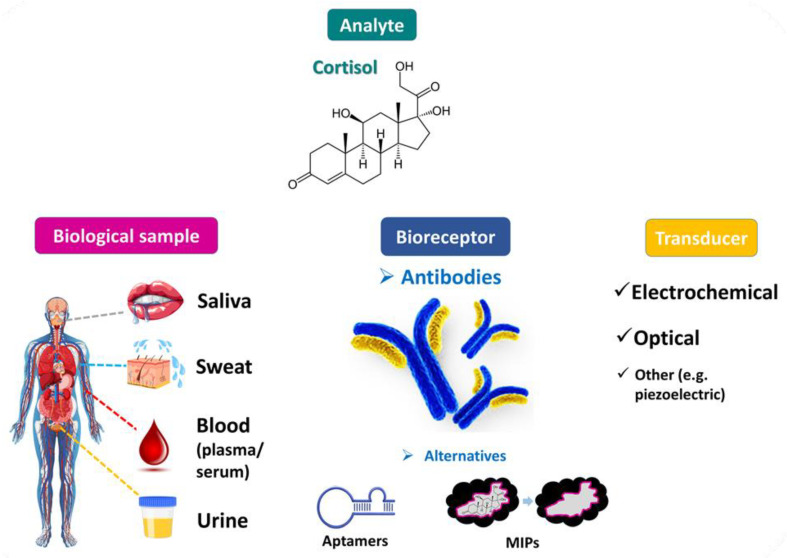
Cortisol has been detected in various biological fluids, and cortisol levels may serve as a valuable biomarker (e.g., of stress). Cortisol determination has been achieved mainly with analytical methods based on specific cortisol binders and especially anti-cortisol antibodies. Cortisol immunosensors based on different signal transduction principles are expected to be eventually commercialized and serve as an easy-to-handle, reliable tool for point-of-care clinical analysis of cortisol.

At present, routine analysis of biological samples for cortisol monitoring is only performed in lab settings [114]. Further research is needed before cortisol immunosensors have become fully commercialized and available to clinical and self-monitoring applications. A first challenge of such efforts would be to perform large validation studies so as to ensure that the analytical characteristics of the immunosensors developed are of high quality. Although there is always space for improvement, sensitivity/LOD is usually not a problem, while specificity and/or simplicity of production and cost-related issues associated with the anti-cortisol antibodies, which are inherent in all immunochemical analytical techniques, might be addressed by antibodies’ replacement with aptamers or MIPs. However, special attention should be paid to the validation of repeatability/reproducibility [115] as well as operational stability, especially when complex biological samples, such as blood plasma/serum are to be analyzed (which may affect integrity and deteriorate functioning of electrodes in electrochemical immunosensors). Conditions of reusability as well as storage stability/durability/life span are also issues to be studied. Wearable immunosensors for detection of cortisol, mainly in sweat samples, seem to provide exciting prospects for further progress in the field, but particular aspects have to be addressed in the years to come [116]. These aspects include accurate sample collection, potential toxicity and biocompatibility of sensor materials, and appropriate power supply of the flexible electronics these sensors require, while data processing and communication constitute a separate research field, which may be further elaborated and improved. Last, but not least, detailed and thorough knowledge of the biological and chemical characteristics of the samples to be analyzed along with deep insight in cortisol physiology and partitioning/kinetics/dynamics of the hormone in different compartments/fluids of the organism in normal and disease states would nicely supplement research in the field (Figure 3), and relevant studies may be performed in parallel. To achieve this, close collaboration among clinicians, physicists, chemists, bioscientists, and engineers is a prerequisite.

## 4. Conclusions

Most reported cortisol immunosensors are currently at the proof-of-concept stage, and further research is necessary before the most appropriate among them could eventually become commercially available. Provided that all issues requiring further elucidation and thorough validation, as discussed in the present work, can be resolved, cortisol immunosensors will be an invaluable analytical tool that will enrich and expand the capabilities of the existing methodology, thus offering exceptional prospects in the field of clinical analysis of cortisol.

## Figures and Tables

**Table 1:** Electrochemical cortisol immunosensors.

Immunoassay Principle	Signal Transduction Principle	Biological Sample	Range/LoD	Reference
Noncompetitive (Direct binding of cortisol to an anti-cortisol Ab ^1^, immobilized onto gold microelectrodes)	Cyclic voltammetry	Buffer	10 pM–500 nM/ 1 pM	[48]
Noncompetitive (Direct binding of cortisol to an anti-cortisol Ab, immobilized onto silver/silver oxide (Ag/AgO)—polyaniline nanocomposites)	Cyclic voltammetry	Buffer	1 pM–1 μM 0.64 pM	[49]
Noncompetitive (Direct binding of cortisol to an anti-cortisol Ab co-immobilized with HRP on the sensor electrode)	Amperometry	Buffer	10^−7^–10^−5^ M	[33]
Competitive (Competition between free cortisol and a BSA-cortisol conjugate immobilized onto magnetic beads for binding to an anti-cortisol Ab labeled with silver nanoclusters (AgNCs)	Photoelectrochemistry	Saliva	0.0001–100 ng/mL/ 0.06 pg/mL	[50]
Noncompetitive (Direct binding of cortisol to an anti-cortisol Ab immobilized on polyaniline-modified graphene electrodes)	Electrochemical impedance spectroscopy	Saliva (canine)	0.0005–50 μg/mL/ 3.57 fg/mL	[37]
Noncompetitive (Direct binding of cortisol to an anti-cortisol Ab labeled with ferrocene-tags and immobilized on a modified tin-doped indium oxide electrode)	Cyclic voltammetry; square wave voltammetry	artificial saliva and zebrafish whole-body	0.001–50 ng/mL/ 1.03 pg/mL	[51]
Noncompetitive (Direct binding of cortisol to an anti-cortisol Ab immobilized on a AuNP/MoS_2_/AuNP–modified screen-printed electrode)	Differential pulse voltammetry	Saliva	0.5–200 nM/ 0.11 nM	[52]
Noncompetitive (Direct binding of cortisol to an anti-cortisol Ab covalently immobilized on NiO thin film/ITO ^2^ electrodes)	Cyclic voltammetry, Differential pulse voltammetry	Saliva	1 pg/mL–10 μg/mL/ 0.32 pg/mL	[53]
Noncompetitive (Direct binding of cortisol to an anti-cortisol Ab covalently immobilized onto micro-Au electrodes)	Electrochemical impedance spectroscopy	Saliva	1 pg/mL–10 ng/mL/ 0.87 ± 0.12 pg/mL	[54]
Noncompetitive (Direct binding of cortisol to an anti-cortisol Ab co-immobilized with BSA on glassy carbon electrodes that had been coated with tin disulfide nanoflakes)	Cyclic voltammetry, differential pulse voltammetry	Saliva	100 pM–100 μM/ 100 pM	[55]
Competitive (Competition between an ALP-labeled cortisol conjugate and free cortisol for binding to an anti-cortisol Ab indirectly immobilized on disposable graphite screen-printed electrodes)	Square wave voltammetry	Saliva	0.5–55.1 ng/mL/ 1.7 ng/mL	[57]
Noncompetitive (Direct binding of cortisol to an anti-cortisol Ab immobilized on electroreduced graphene oxide deposited on screen-printed electrodes)	Electrochemical chronoamperometry	Saliva, sweat	0.1 ng/mL	[56]
Noncompetitive (Direct binding of cortisol to an anti-cortisol Ab covalently immobilized on reduced graphene oxide channels between two planar electrodes)	Resistance	Human saliva and buffer solution of rat adrenal gland acute slices	10 pg/mL	[58]
Noncompetitive (Direct binding of cortisol to an anti-cortisol Ab immobilized on Au-substrates modified with ZnO nanostructures (1D nanorods, 2D nanoflakes))	Cyclic voltammetry	Saliva	1 pM	[59]
Noncompetitive (Direct binding of cortisol to an anti-cortisol Ab covalently immobilized on microfabricated interdigitated microelectrodes)	Cyclic voltammetry	Saliva	10 pg/mL–100 ng/mL/ 10 pg/mL	[60]
Competitive (Competition between a GOD ^3^–cortisol conjugate and free cortisol for binding to an anti-cortisol Ab immobilized on platinum electrodes; lateral and vertical fluid control mechanisms were integrated in the sensor)	Amperometry	Saliva	0.1–10 ng/mL	[61]
Noncompetitive (Direct binding of cortisol to an anti-cortisol Ab immobilized on gold microelectrode arrays)	Electrochemical impedance spectroscopy	Saliva and interstitial fluid	1 pM–100 nM	[63]
Competitive (Competition between free cortisol and a cortisol analog covalently immobilized on single-walled carbon nanotubes and free cortisol for binding to an anti-cortisol Ab)	Resistance/conductance	Saliva	1 pg/mL–10 ng/mL/ 1 pg/mL	[62]
Competitive (Competition between cortisol immobilized on naflon pretreated glassy carbon electrodes and free cortisol for binding to a biotinylated anti-cortisol Ab; detection was performed via reaction with HRP–streptavidin)	Electrochemical impedance spectroscopy, cyclic voltammetry	Plasma	0.1–1000 ng/mL/ 0.05 ng/mL	[65]
Noncompetitive (Direct binding of cortisol to an anti-cortisol Ab co-immobilized with GOD on gold electrodes)	Amperometry	Plasma (fish)	1.25–200 ng/mL	[66]
Noncompetitive (Direct binding of cortisol to an anti-cortisol Ab, immobilized on interdigitated gold microelectrodes)	Cyclic voltammetry	Plasma	10 pg/mL–500 ng/mL/ 10 pg/mL	[67]
Competitive (Competition between an ALP-labeled cortisol conjugate and free cortisol for binding to an anti-cortisol Ab covalently immobilized on gold electrodes.	Amperometry	Serum	0–250 ng/mL/ 13.4 ng/mL	[64]
Competitive (Competition between an ALP-labeled cortisol conjugate and free cortisol for binding to an anti-cortisol Ab immobilized through protein A on magnetic particles; the immunocomplexes formed were trapped on the surface of screen-printed electrodes with a small magnet and ALP activity was monitored)	Differential pulse voltammetry	Serum	5 × 10^−3^–150 ng/mL/ 3.5 pg/mL	[69]
Competitive (Competition between an HRP-labeled cortisol conjugate and free cortisol for binding to an anti-cortisol Ab immobilized on gold electrodes functionalized with a AuNP–protein G–DTBP ^4^ scaffold)	Square wave voltammetry	Buffer, Serum	50–2,500 pg/mL/ 16 pg/mL	[68]
Noncompetitive (Direct binding of cortisol and cortisone (transformed into cortisol via the enzyme 3α-hydroxysteroid dehydrogenase) to an anti-cortisol Ab immobilized on gold nanowires/working electrodes)	Square wave voltammetry	Buffer, Serum	10–80 μM	[70]
Noncompetitive (Direct binding of cortisol to an anti-cortisol Ab immobilized on L-cys ^5^–AuNPs–MXene-modified electrodes)	Amperometry	Artificial Sweat	5–180 ng/mL/ 0.54 ng/mL	[41]
Noncompetitive (Direct binding of cortisol to an anti-cortisol Ab immobilized on the surface of flexible screen-printed electrodes coated with AuNPs)	Differential pulse voltammetry	Sweat	7.47 nM	[42]
Noncompetitive (Direct binding of cortisol to an anti-cortisol Ab, immobilized on a flexible electrode prepared on polydimethylsiloxane modified with multiwalled carbon nanotubes and AuNPs)	Cyclic voltammetry, differential pulse voltammetry	Sweat	1 fg/mL–1 μg/mL/ 0.3 fg/mL	[43]
Competitive (Competition between an HRP–cortisol conjugate and free cortisol for binding to an anti-cortisol antibody immobilized on graphene-based electrode)	Amperometry	Sweat	0.43–50.2 ng/mL	[44]
Noncompetitive (Direct binding of cortisol to an anti-cortisol antibody immobilized on a conductive carbon yarn functionalized with ellipsoidal Fe_2_O_3_ particles)	Cyclic voltammetry	Sweat	1 fg/mL–1 μg/mL/ 0.005 fg/mL	[73]
Noncompetitive (Direct binding of cortisol to an anti-cortisol Ab immobilized on MoS_2_ sheets integrated into a nanoporous flexible electrode system)	Electrochemical impedance spectroscopy	Sweat	1–500 ng/mL/ 1 ng/mL	[74]
Noncompetitive (Direct binding of cortisol to an anti-cortisol Ab immobilized on ZnO thin film deposited on a flexible nanoporous polyamide membrane; room temperature ionic liquids were employed to enhance sensor stability)	Electrochemical impedance spectroscopy	Sweat	10–200 ng/mL/ 10 ng/mL	[75]
Noncompetitive (Direct binding of cortisol to an anti-cortisol Ab immobilized on a ZnO thin film deposited on a flexible nanoporous polyamide membrane)	Electrochemical impedance spectroscopy	Sweat	10–200 ng/mL/ 1 ng/mL	[76]
Noncompetitive (Direct binding of cortisol to an anti-cortisol Ab, covalently immobilized on a gold microelectrode array)	Electrochemical impedance spectroscopy	Interstitial fluid	1 pM–100 nM	[71]
Competitive (Competition between free cortisol and an HRP–cortisol conjugate for binding to an anti-cortisol antibody immobilized on a platinum electrode)	Amperometry	Dialysates of extracellular fluid of animal brain, amygdala region (sheep)	0–100 ng/mL (in vitro measurement)	[72]
Competitive (Competition between HRP–cortisol conjugate and free cortisol for binding to an anti-cortisol antibody immobilized on platinum electrodes)	Potentiometry	Dialysates of animal circulating blood (sheep, cattle, rat)	0.3 μg/100 mL	[34]

^1^ Ab: antibody. ^2^ ITO: indium tin oxide. ^3^ GOD: glucose oxidase. ^4^ DTBP: dimethyl 3,3′-dithiobispropionimidate.2HCl. ^5^ L-cys: L-cysteine.

**Table 2:** Optical cortisol immunosensors.

Immunoassay Principle	Signal Transduction Principle	Biological Sample	Range/LoD	Reference
Noncompetitive (Direct binding of cortisol to an anti-cortisol Ab immobilized on a D-shaped, gold-coated silica optical fiber)	Surface plasmon resonance (SPR)	Buffer	0.01–100 ng/mL/ 1.46 ng/mL	[81]
Noncompetitive (Direct binding of cortisol to an anti-cortisol Ab immobilized on a plastic optical fiber coated with gold–palladium allοy)	SPR	Buffer	1 pg/mL	[82]
Competitive (Competition between a fluorescently labeled BSA–cortisol conjugate and free cortisol for binding to an anti-cortisol Ab immobilized on glass substrate coated with gold)	Metal-enhanced fluorescence (MEF)	Buffer	0.02 μg/mL	[83]
Noncompetitive (Direct binding between of cortisol to an anti-cortisol Ab or aptamer, immobilized on quantum dots)	Fluorescence quenching	Saliva	1 nM (aptamer-based) 100 pM (Ab-based)	[84]
Lateral flow–type Competitive (Competition between a BSA–cortisol conjugate immobilized on the strip and free cortisol for binding to a Cy3-labeled anti-cortisol Ab)	Fluorescence (detected with a smartphone-linked reader)	Saliva	0.1 ng/mL	[85]
Competitive (Competition between an HRP-labeled cortisol conjugate and free cortisol for binding to an anti-cortisol Ab indirectly immobilized on PDMS microfluidic channel)	Colorimetry	Saliva	0.01–20 ng/mL/ 18 pg/mL	[86]
Lateral flow–type Competitive (Competition between an HRP-labeled cortisol conjugate and free cortisol for binding to an anti-cortisol Ab immobilized on the strip)	Chemiluminescence (detected through a smartphone camera)	Saliva	0.3–60 ng/mL/ 0.3 ng/mL	[87]
Lateral flow–type Competitive (Based on europium fluorescent particle conjugates)	Fluorescence (Detected with a cassette reader transferring results through a Bluetooth device, manufactured by Oasis Diagnostics)	Saliva	0.91 ng/mL	[88]
Competitive (Competition between a cortisol analogue, hydrocortisone 3-(O-carboxymethyl)oxime, covalently immobilized on gold surface and free cortisol for binding to an anti-cortisol Ab)	SPR	Saliva	10 ppt–100 ppb/ 38 ppt	[89]
Competitive (Competition between a BSA–cortisol conjugate immobilized on a disposable disk chip and free cortisol for binding to an ALP-labeled anti-cortisol Ab)	Chemiluminescence	Saliva	0.4–11.3 ng/mL	[90]
Competitive (Competition between an in-house prepared cortisol conjugate immobilized on a gold sensor surface and free cortisol for binding to the anti-cortisol Ab; a secondary Ab was used for signal increase)	SPR	Saliva	91–934 pg/mL/ 49 pg/mL	[79]
Noncompetitive (Direct binding of cortisol to an anti-cortisol Ab covalently immobilized on the polycarboxylate hydrogel–coated sensing surface)	SPR	Saliva, urine	3 μg/L	[78]
Competitive (Competition between a BSA–cortisol conjugate immobilized on the SPR-sensor surface and free cortisol for binding to a monoclonal anti-cortisol Ab)	SPR	Saliva, buffer	1.0 ng/mL	[77]
Competitive (Competition between cortisol analogues, i.e., suitably prepared cortisol–ssDNA conjugates, and free cortisol for binding to a biotinylated anti-cortisol Ab immobilized on streptavidin-coated particles)	Particle mobility (detected through dark field microscopy)	Blood plasma (filtered or microdialysis-sampled)	High nM–low μM	[91]
Competitive (Competition between a BSA–cortisol conjugate immobilized on paper and free cortisol for binding to gold nanoparticles loaded with the anti-cortisol Ab)	Color	Blood serum	21.5 μg/dL	[92]

## Data Availability

Not applicable.
